# Neonatal abstinence syndrome is a potential cause of low TREC copy number

**DOI:** 10.1186/s13223-021-00617-3

**Published:** 2021-11-02

**Authors:** Adil Adatia, Ling Ling, Pranesh Chakraborty, Lauren Brick, Rae Brager

**Affiliations:** 1 Division of Clinical Immunology and Allergy, Department of Medicine, McMaster University, Hamilton, ON Canada; 2 Department of Medicine, University of Alberta, Edmonton, AB Canada; 3 Newborn Screening Ontario, Children’s Hospital of Eastern Ontario, Ottawa, ON Canada; 4 Department of Pediatrics, Children’s Hospital of Eastern Ontario, Ottawa, ON Canada; 5 Division of Rheumatology, Immunology, and Allergy, Department of Pediatrics, McMaster Children’s Hospital, McMaster University, Hamilton, ON Canada

**Keywords:** SCID, Primary immunodeficiency, TRECs, Opiates, Neonatal abstinence syndrome

## Abstract

Severe combined immunodeficiency (SCID) is a rare genetic condition characterized by significant T cell lymphopenia and impaired T cell function. Many jurisdictions use the quantitation of T cell receptor excision circles (TRECs) to screen for SCID in newborns, but false positives may be seen in several conditions. We report 3 newborns with neonatal abstinence syndrome who presented with decreased TREC copy number.

Severe Combined Immunodeficiency (SCID) is a rare congenital disorder characterized by profound T cell lymphopenia and poor T cell function. T cell Receptor Excision Circles (TRECs), a by-product of T cell receptor recombination, is a marker of thymic output of naïve T cells and can be quantified using Guthrie Card dried blood samples obtained from newborns. TREC quantification using real-time quantitative PCR has been widely integrated into newborn screening programs worldwide as a screening test for SCID [1]. The Canadian province of Ontario added TREC quantification to its newborn screening (NBS) program in 2013.

TREC quantification has excellent sensitivity but lower specificity for SCID. The positive predictive value is 0.8–11.2% for SCID and 18.3–81% for T cell lymphopenia [2]. Various medical conditions have been reported to cause low TREC values in the absence of defined immune deficiency syndromes. The most common secondary causes of low TREC copy number are prematurity, and cardiac, gastrointestinal, or multisystem anomalies, but many cases remain idiopathic. The rate of these false positive results is highly sensitive to the threshold chosen to identify abnormal results [3].

We report three neonates who were referred by the Ontario NBS program to a tertiary care Pediatric centre in Hamilton, Ontario for low TREC copy number between 2014 and 2017 who had neonatal abstinence syndrome (Table 1). Repeat TREC enumeration and T cell immunophenotyping on day 13 of life were normal, and all patients had normal 22q11.2 FISH and normal serum purine concentrations. The patients had no other medical comorbidities to explain their transient T cell lymphopenia. The mothers of the neonates tested negative for HIV and had no history of immunosuppressant medication use. Patients 1 and 3 were preterm, but late preterm newborns typically have very similar TREC copy numbers to those born at term [4]. Patients 2 and 3 had low birthweight, which was attributed to maternal recreational drug use.

**Table 1:** Clinical and immunological characteristics of 3 newborns presenting with abnormal TREC copy number

	GA (weeks)	Sex	Mode of delivery	Birth weight (g)	Antenatal exposures	Medical history	TREC (number/µL)		T cell counts^a^ (× 10^9^/L)
							Initial	Repeat	
1	36	M	CS	2961	Methadone Marijuana	Neonatal abstinence syndrome	66	385	CD3 + 3.22 CD3 + CD4 + 2.53 CD3 + CD8 + 0.78
2	37	F	CS	1590	Methadone Heroin	Neonatal abstinence syndrome Symmetrical IUGR	46	299	CD3 + 6.64 CD3 + CD4 + 3.85 CD3 + CD8 + 2.79
3	36 + 6	F	CS	2000	Methadone Heroin	Neonatal abstinence syndrome Symmetrical IUGR	51	253	CD3 + 7.74 CD3 + CD4 + 5.93 CD3 + CD8 + 1.69

Patients 2 and 3 were diachorionic diamnionic twins. All 3 newborns had their second TREC copy number and T cell counts measured on the 13th day of life

*CS* caesarean section, *GA* gestational age at birth, *IUGR* intrauterine growth restriction, *SVD* spontaneous vaginal delivery, *TREC* T cell receptor excision circle

^a^Normal values for T cell counts: CD3 + 1.5–5.0 × 10^9^/L, CD3 + CD4 + 0.90–3.60 × 10^9^/L, CD3 + CD8 + 0.30–1.40 × 10^9^/L

Opioids including methadone and heroin readily cross the placental barrier [5]. We therefore posit that maternal opioid use during pregnancy may cause or contribute to transient T cell lymphopenia in the newborn. The specific mechanisms by which this could occur are unclear, but perinatal stress associated with neonatal abstinence syndrome seems a plausible explanation. Data from animal models also show that opioids may directly affect T cells lymphocytes. Opioids can cause thymic and splenic atrophy [6], significantly decreased thymus cellularity [6, 7], and decreased mitogen responses [8]. Flow cytometry of thymocytes from mice exposed to morphine showed depletion of double negative T cells [7], which would be expected to cause decreased TREC numbers since this cell population has not undergone V(D)J recombination.

In the USA, antepartum opioid use is estimated at nearly 6 per 1000 hospital births [5], but the rate of low TRECs is approximately 1 per 1000 newborns screened, many of whom have alternate explanations for capture [3]. We speculate that this discrepancy is multifactorial, including the potential need for chronic, perhaps high-dose opioid exposure during the third trimester, leading to neonatal abstinence syndrome, and additional genetic or environmental factors to contribute to transient neonatal T cell lymphopenia. There may also be contributions from other recreational drug exposures.

The potential for maternal opioid use to decrease TREC values is of significant public health import. Jurisdictions such as ours with a higher rate of illicit drug use may have a higher number of false positive screening results causing unnecessary follow up testing, cost, and parental psychological stress. During the period of study, the Ontario NBS program used a threshold of less than 75 copies/µL to identify abnormal results. Adjusting the TREC cutoff to < 25 copies/µL, which is used by numerous states in the USA [3], would reclassify all 3 cases as negative for SCID. Importantly, lowering TREC thresholds to 20–25 copies/microliter does not appear to increase the number of false negative results [3, 9].

In summary, we report a series of 3 neonates presenting with neonatal abstinence syndrome and low TREC copy numbers in the absence of SCID. To our knowledge, this is the first report associating maternal opioid use with transiently low T cells in newborns. We hypothesize that antenatal opioid exposure may contribute to T cell lymphopenia in the newborn period and may be the sole cause in a small subset of cases. The possibility of broad immune effects from antenatal opioid exposure based on animal data indicate that follow-up of infants with low TREC values should be continued even when the possible etiology is NAS. Given that SCID is a rare condition and TREC enumeration is a recent addition to NBS in many areas, additional non-SCID causes of low TREC numbers are likely to be identified in the future. Consideration of these aetiologies is important when evaluating individual cases and for the optimization of nascent SCID screening programs.

## Data Availability

All data are contained within the manuscript.

## Abbreviations

CS: Caesarean section
GA: Gestational age at birth
IUGR: Intrauterine growth restriction
NBS: Newborn screening
SVD: Spontaneous vaginal delivery
SCID: Severe combined immunodeficiency
TREC: T cell receptor excision circle
